# Neurodevelopmental Disruptions in Children of Preeclamptic Mothers: Pathophysiological Mechanisms and Consequences

**DOI:** 10.3390/ijms25073632

**Published:** 2024-03-24

**Authors:** Andrea González-Rojas, Martina Valencia-Narbona

**Affiliations:** Laboratorio de Neurociencias Aplicadas, Escuela de Kinesiología, Facultad de Ciencias, Pontificia Universidad Católica de Valparaíso, Avenida Brasil 2950, Valparaíso 2340025, Chile; martina.valencia@pucv.cl

**Keywords:** preeclampsia, neurodevelopment, offspring, cerebral palsy, autism, spectrum disorder, attention deficit hyperactivity disorder, pathophysiological mechanisms

## Abstract

Preeclampsia (PE) is a multisystem disorder characterized by elevated blood pressure in the mother, typically occurring after 20 weeks of gestation and posing risks to both maternal and fetal health. PE causes placental changes that can affect the fetus, particularly neurodevelopment. Its key pathophysiological mechanisms encompass hypoxia, vascular and angiogenic dysregulation, inflammation, neuronal and glial alterations, and disruptions in neuronal signaling. Animal models indicate that PE is correlated with neurodevelopmental alterations and cognitive dysfunctions in offspring and in humans, an association between PE and conditions such as cerebral palsy, autism spectrum disorder, attention deficit hyperactivity disorder, and sexual dimorphism has been observed. Considering the relevance for mothers and children, we conducted a narrative literature review to describe the relationships between the pathophysiological mechanisms behind neurodevelopmental alterations in the offspring of PE mothers, along with their potential consequences. Furthermore, we emphasize aspects pertinent to the prevention/treatment of PE in pregnant mothers and alterations observed in their offspring. The present narrative review offers a current, complete, and exhaustive analysis of (i) the pathophysiological mechanisms that can affect neurodevelopment in the children of PE mothers, (ii) the relationship between PE and neurological alterations in offspring, and (iii) the prevention/treatment of PE.

## 1. Introduction

Preeclampsia (PE) is one of the most serious complications during pregnancy, with a prevalence ranging from 2% to 8% of all pregnancies worldwide [1]. PE is a multisystem disorder characterized by high blood pressure, which occurs after 20 weeks of gestation and is accompanied with proteinuria (≥300 mg of protein in the urine in 24 h). It can also manifest with failure in one or more organs or tissues, presenting as a protein/creatinine ratio ≥ 0.3 (mg/dL), hemolysis, elevated liver enzymes, a low platelet count (<100,000/μL), an abnormal Doppler ultrasound, or neurological or visual symptoms leading to fetal growth restriction [2,3,4,5,6,7,8].

PE can be classified, according to gestational age at diagnosis or delivery, as early (<34 weeks) or late PE (≥34 weeks) [7]. Although early-onset PE has a lower prevalence than late-onset PE, early-onset PE is associated with greater maternal morbidity, perinatal death, and neonatal morbidity [9].

There are several risk factors that could influence the development of PE, including family history of PE; age older than 35 years; multiple pregnancies; in vitro fertilization; previous placental abruption; previous intrauterine fetal growth restriction; molar pregnancy; systemic lupus erythematosus; maternal smoking; trisomy of chromosome 13; and maternal comorbidities such as chronic hypertension, kidney disease, diabetes mellitus, and/or obesity [3,5,10].

PE has the potential to cause complications during pregnancy. Approximately 25% of pregnant women may develop the HELLP syndrome (hemolysis, elevated liver enzymes, and low platelet count) or atypical PE, which causes increased liver enzymes, thrombocytopenia, and low platelet levels [7,11,12]. Eclampsia is another severe complication of PE, which may manifest in 0.3% of live births; however, the incidence rates vary significantly based on maternal risk factors, economic disparities, and access to quality prenatal care [13]. It often manifests alongside tonic–clonic seizures in pregnant women with PE, despite the absence of other causes of seizures during the gestational period, the presence of which would cause even greater multisystem deterioration in the offspring [14,15,16,17]. Hypertension during pregnancy accounts for one of the main causes of maternal death, reaching 25% in developing countries and 16% in industrialized countries [15,18].

In addition to the complications that PE can cause in a mother, a close relationship has been described between the intrauterine environment and the alterations observed in the children of mothers with PE [7,9,18,19,20,21]. In this regard, during a normal pregnancy—specifically, in the early phase of placentation—trophoblastic cells invade the wall of maternal uterine spiral arteries, remodeling them and transforming them into vessels with a large diameter and low resistance to favor increased blood flow, thus aiding in fetal development [18,20,22,23,24,25,26]. In PE, abnormal placentation occurs, and placental hypoperfusion induces the release of placental factors into the maternal circulation, leading to generalized endothelial dysfunction and subsequently resulting in systemic vascular dysfunction, highlighting defects in angiogenesis at the brain level [5,24,25,26,27,28,29]. In early-onset PE, the inability to remodel the wall of spiral arteries has been observed, maintaining narrow arteries and leading to high pressure, hypoperfusion, placental hypoxia, and ischemia [9,20,21]. The reduced blood flow observed in early-onset PE directly affects placental development, causing placental dysfunction that can induce fetal growth restriction [9,20,21]. Therefore, the associated pathophysiological changes, such as placental ischemia, hypoxia, and hypoperfusion, combined with the proinflammatory state characterizing PE, result in a decrease in nutrient transfer to the fetus, which could trigger alterations in the central nervous system (CNS). This has been observed in both animal models and humans, especially in the first-born offspring of mothers with PE (PE-F1) [30,31].

Evidence suggests a strong association between PE and offspring alterations in both neurodevelopment and neurocognitive functions in PE-F1 [6,32,33]. In humans, a strong association has been observed between PE and the presence of CNS complications, such as cerebral palsy (CP) [34], autism spectrum disorder (ASD) [32,35], attention deficit hyperactivity disorder (ADHD) [35], depression [36], decreased intelligence quotient (IQ) [20], cerebrovascular accident [20], and epilepsy [6,32]. Furthermore, in humans (as observed in rodents), PE-F1 individuals exhibit a smaller head circumference compared to offspring of normotensive mothers [37,38], along with neuroanatomical alterations (enlargement of certain brain regions and vessels), determined through magnetic resonance imaging (MRI) in individuals aged 7 to 10 years [37].

In rodents, the mid- and late stages of gestation (embryonic days 14.5–20.5 in rodents; weeks 20–40 in humans) have been identified as critical periods for fetal brain development in utero and, thus, are highly susceptible to developing CNS alterations and the onset of PE [39]. Animal models have shown that PE-F1 animals exhibit deficiencies in spatial working memory and exploratory behavior [37,39]. Additionally, they have smaller relative and absolute brain volumes in areas such as the cerebral cortex, occipital lobe, and entorhinal cortex, which may result in cognitive function deficiencies [37,39].

Therefore, PE has not only potentially life-threatening consequences for mothers but also serious implications for the neurodevelopment of their children. The direct or indirect impact of PE on the developing child’s CNS could trigger significant neurological complications during growth. However, the alterations that occur in offspring as a result of maternal PE have not yet been fully described or systematized. Therefore, the aim of this narrative literature review is to describe the pathophysiological mechanisms related to the neurodevelopmental alterations observed in the offspring of preeclamptic mothers, as well as their potential consequences. Finally, we emphasize aspects pertinent to the prevention and treatment of PE in pregnant mothers and the alterations observed in their offspring.

## 2. Physiopathological Mechanisms That May Affect Neurodevelopment in Children of Preeclamptic Mothers

**Figure 1:**
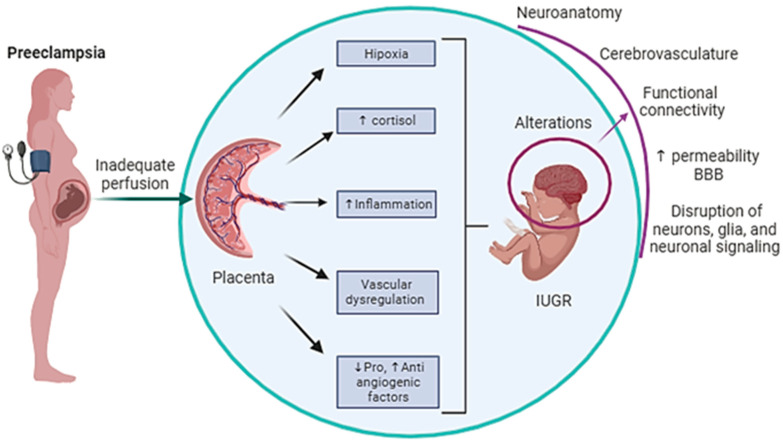
Physiopathological mechanisms that may affect neurodevelopment in children of preeclamptic mothers. Placental alterations caused by PE promote increased inflammation and cortisol, conditions of hypoxia, vascular dysregulation, and alterations in pro- and anti-angiogenic factors. These conditions induce an unfavorable intrauterine environment for the fetus. This adverse environment is associated with neuroanatomical changes; cerebrovascular alterations; connectivity disruptions; changes in the permeability of the BBB; and disruptions in neurons, glia, and neuronal signaling. Ultimately, these alterations impact the neurodevelopment of the child. Abbreviations: PE, preeclampsia; IUGR, intrauterine growth restriction; BBB, blood–brain barrier; ↑, increase; ↓, decrease.

Considering that fetal development is a critical period, an adverse fetal environment such as that occurring in PE can affect neurodevelopmental disorders [40]. The possible pathophysiological mechanisms of neurodevelopmental alterations include hypoxia [32,40,41,42,43,44,45,46], impairment of vascular function [37,39,40,41,43,47,48,49], disturbance in pro- and anti-angiogenic factors [37,43,44,45,48,49,50], inflammation [32,41,43,44,45,46,51], exposure to elevated levels of glucocorticoids [47,52], and abnormal placentation [37,39,40,42] (Figure 1), as described below.

### 2.1. Hypoxia and Excess Cortisol

Hypoxia is a stressor that induces an adverse intrauterine environment for the developing fetus and has been associated with cardiovascular and metabolic pathologies [53]. Notably, prenatal exposure to PE can lead to intrauterine fetal hypoxia, which confers critical long-term consequences for future infant neurodevelopment, where the constriction of blood vessels related to PE generates hypoxia in the placental environment [44,54]. In fact, animal models have revealed that damage from hypoxia could lead to permanent changes in brain structure [55]. A possible mechanism for the development of hypoxia during preeclamptic pregnancy is that increased blood pressure leads to uteroplacental dysfunction through reducing the supply of oxygen and nutrients to the fetus, resulting in a progressive state of oxidative stress (OS) [40,41].

An increase in OS can induce the release of syncytiotrophoblast proteins into the maternal bloodstream (to enhance circulation), promoting impaired vascular and immune responses, exaggerating basal systemic inflammation, and causing changes in the vascular endothelium [41,42,44,50,52]. Changes in the vascular endothelium can be triggered by circulating factors in the blood of women with PE, leading to OS. These factors include reactive oxygen species (ROS) produced by neutrophils (oxLDL); autoantibodies agonistic to angiotensin receptors (AT1-AA); free fetal hemoglobin (HbF); circulating xanthine oxidase (XO); and cytokines, specifically TNF-α. Additionally, in endothelial cells, certain enzymatic systems such as the electron transport chain, NADPH oxidases, and cyclooxygenases can generate superoxide anion (O_2_^•−^). This anion can increase the expression of arginase II and asymmetric dimethylarginine (ADMA); cause tetrahydrobiopterin (BH4) cofactor loss; and uncouple endothelial nitric oxide synthase (eNOS), resulting in O_2_^•−^ generation. Nitric oxide can react with O_2_^•−^ to form peroxynitrite (ONOO^−^), a potent oxidant that can damage DNA via nitrated proteins. Additionally, ONOO^−^ can inhibit eNOS activity and impact endothelium-dependent vasodilation. Furthermore, ROS can also negatively regulate calcium-activated potassium channels KCa2.3 and KCa3.1, which are crucial for vasodilation [56,57,58]. Due to the above, a placental ischemic environment is subsequently generated through reducing blood flow in the fetoplacental unit, leading to hypoxia [32,40,41,42,43,44,45,46]. The hypoxic placenta may secrete altered RNA, along with increased expression levels of hypoxia-inducible factor 1 alpha (HIF-1α) [32,42,59] and placental tumor necrosis factor-alpha (TNF-α) [46,59] as well as an increase in hematocrit [46], which may favor the effects of physiological stress induced by PE and placental hypoxia [32,59]. Placental ischemia and the decrease in antioxidants that occurs in PE decrease nitric oxide synthase 3 (NOS3), thereby decreasing the production of nitric oxide (NO), which participates in angiogenic and vascular regulation, causing a negative impact on the brain of PE-F1 [32].

This fetal–placental stress due to hypoxia can disrupt the fetal hypothalamic–pituitary–adrenal (HPA) axis, promoting cardiovascular, metabolic, and immune system alterations that may result in fetal growth restriction [6,53]. In this context, the HPA axis during pregnancy regulates the differentiation and maturation of organs such as the lungs, liver, and kidneys, as well as the regulation of metabolism (lipolysis, glycogenolysis, and protein catabolism) through the synthesis of glucocorticoids, especially cortisol [60,61,62,63,64]. Under conditions of chronic stress, the HPA axis secretes abnormally high amounts of cortisol, inducing hyperglycemia, immune system suppression, increased lipid disposition, bone loss, and arterial hypertension [65,66,67].

Another key element in fetal hypoxia in PE may be peripheral adipose tissue (PAT). PAT develops and matures in the final stage of gestation, having metabolic and endocrine functions, storing and releasing lipids, and promoting the secretion of leptin [68]. Fetal hypoxic stress may play a role in altered fetal programming due to the influence of leptin, a hormone that regulates appetite in adulthood. Leptin can regulate cortisol secretion and, therefore, the fetal response to hypoxia, hence its relevance [53].

Uteroplacental ischemia observed in preeclamptic pregnancies leads to cerebrovascular changes, a greater disruption of the blood–brain barrier (BBB), and microhemorrhages in the fetal brain, contributing to both short- and long-term neurological development complications associated with PE-F1 [41,46]. In this regard, both acute and chronic fetal hypoxia have been linked to an increased risk of developing ASD [41] and schizophrenia, associated with elevated levels of adenosine [59]. High levels of cerebral adenosine hyperactivate adenosine A1 receptors (A1R), which mediate neurotoxicity in the early stages of brain development, could thus negatively affect brain development [59].

### 2.2. Vascular and Angiogenic Dysregulation

Abnormal vascular growth and function are determinants in the pathogenesis of PE [40,41,43,44,47,48,49]. In this regard, a disruption in vascularization could negatively impact neuronal development and vice versa [37]. In general, neuronal and cerebral vascular development are closely related, and both use the same family of ligands and receptors to regulate important processes that are altered in PE, such as survival, proliferation, angiogenesis, cell organization, cerebral vasculature, and endothelial and neuronal “vascular pruning” processes [69]. PE is associated with alterations in placental pro- and anti-angiogenic factors [43,47,48,50], such as vascular endothelial growth factor (VEGF) [44,46,49], placental growth factor (PGF) [37,44,47,49], soluble fms-like tyrosine kinase 1 receptor (sFlt-1) [37,44,45,47,49], and soluble endoglin (sEng) [70,71,72].

VEGF is part of the endothelial growth factor family consisting of VEGF-A, VEGF-B, VEGF-C, VEGF-D, VEGF-E, VEGF-F, PGF, and endocrine gland-derived VEGF (EG-VEGF) [73,74]. VEGF interacts with receptor 1 (VEGFR1 (Flt1)) and receptor 2 (VEGFR2 (KDR/FlK1)) to carry out its functions and is a key protein in the neural and vascular development of the brain [28,75]. When binding to VEGF-R2, VEGF regulates the development of morphological features of cerebral blood vessels and vascular permeability [76,77], as well as the dendrogenesis and somatogenesis of Purkinje cells [73,78,79,80,81,82]. For example, given that VEGF is a signal for neuronal migration and a facilitator of the stimulation of action potentials, it has been suggested that a decrease in the expression of this factor—for example, at the cerebellar level—could deregulate the molecular signaling cascades involved in vascular restructuring, synapses, and, therefore, functional processes. In this way, alterations in the development of the neuronal network in this area, which performs both cognitive and motor functions through integrating sensory and motor pathways, affect motor coordination and balance [83,84]. Additionally, the phosphorylation of tyrosine residue 951 (Y951) and tyrosine 1175 (Y1175) by VEGF on VEGF-R2 induces cell migration, proliferation, and angiogenesis [76,85,86].

Reduced concentrations of free circulating VEGF have been observed during clinical PE and before its onset, primarily due to the blocking of VEGF by sFlt1 and sEng, as described below [87,88,89]. Despite this, an increase in VEGF has been observed in PE-F1 mice, which could occur to compensate for the reduced levels of PGF [37]. A well-established and commonly employed method for inducing PE in mothers is through the administration of NG-nitro-L-arginine methyl ester (L-NAME) [90,91,92]. Research from our laboratory evaluated the effect of L-NAME-induced PE on offspring at an ontogenetic stage equivalent to childhood through the cerebellar expression of VEGF and two of its receptors, pY951 VEGF R2 and pY1172 VEGF R2. The results showed a decrease in VEGF in the progeny of both sexes and an increase in phosphorylated VEGFR2 at Y951 only in male offspring. Additionally, a decrease in locomotor learning was observed only in the female offspring of preeclamptic mothers, suggesting that these neurodevelopmental alterations could be occurring (at least in part) due to the decreased VEGF in the cerebellar vermis, the dysregulation of molecular signaling pathways it involves, and the processes it participates in. From these results, it can be inferred that the locomotor alterations evidenced in our study could be mediated by the dysregulation of VEGF expression in the cerebellar vermis of both sexes of the progeny but not in the expression of VEGF receptors (in an activated state) analyzed in the present research. Therefore, future studies are necessary to continue understanding the molecular mechanisms involved in the complex pathophysiology of PE [91].

As stated above, PGF is a proangiogenic factor and a member of the VEGF family [37]. The concentration of PGF increases in the plasma of mothers with normal pregnancies, being associated with fetal growth as well as the arteriolar caliber of the retina in children [93,94,95]. It is highly dependent on the placenta, reflected in higher concentrations in both the umbilical cord blood and amniotic fluid [96,97]. In mothers with early-onset PE, the circulating PGF concentration is low starting from the first trimester, which can have critical consequences for fetal development [48,70,96,97,98,99,100,101]. In a rodent model of Pgf^−/−^, Kay et al., 2019, demonstrated impairments in spatial working memory and exploratory capacity, as well as a decrease in the size of the occipital lobe and entorhinal cortex [37]. Furthermore, alterations in the vascular organization of the retina have been identified, as have changes in the vascularization of the rhombencephalon. Decreased connectivity in the Circle of Willis has also been observed in the same model [102,103]. In a model of PE induced by L-NAME in rats, offspring exhibited reduced cortical thickness, an increased number of glial cells, and decreased neurogenesis in the hippocampus in adulthood [39]. Additionally, alterations in spatial memory and learning were identified [39,104,105]. The above indicates a deficiency in the development of cognitive functions, cerebral neuroanatomy, and cerebral vascularization associated with the absence of PGF, highlighting its fundamental role in this process.

The dysregulation of sFlt-1 may be one of the factors inducing angiogenic and vascular alterations in PE [37,44,45,47,49]. sFlt-1, also known as soluble vascular endothelial growth factor receptor 1 (sVEGFR1), is a receptor produced by the placenta and is an alternatively spliced version of VEGFR1 (Flt1) with antiangiogenic functions that increase in PE [106,107,108]. In PE, sFlt-1 levels rise even before the mother shows clinical symptoms, and these levels are related to the severity of PE [70]. The angiogenic imbalance in PE occurs due to the binding of sFlt-1 to VEGF and PGF, preventing their interaction with endothelial receptors and leading to generalized endothelial dysfunction and systemic vascular dysfunction [108]. Under conditions of hypoxemia and hypoperfused placenta, excess sFlt-1 is produced, favoring its binding to VEGF and PGF in maternal serum and blocking their proangiogenic effects, as well as endothelial cell growth, uterine vessel vasodilation, and placental development. This disrupts angiogenesis and may promote neurodevelopmental alterations in the children of mothers with PE [28,106,107].

Studies on PE and eclampsia, as well as HELLP, have shown that the levels of sEng and sFlt-1 increase during pregnancy and contribute to the creation of a state of placental inflammation and endothelial dysfunction, along with a decrease in offspring birth weight [45,109]. Endoglin (Eng) is a co-receptor for transforming growth factor β1 and β3 (TGF-β1 and TGF-β3, respectively), and its soluble form derived from the placenta is an antiangiogenic factor that could inhibit TGF-β1 signaling in the vasculature and is related to the etiology of PE [70,71,72,110]. sEng negatively affects the binding of TGF-β1 to its receptors and the subsequent signaling cascade, such as eNOS activation and vasodilation, indicating that sEng induces dysregulated TGF-β signaling in vascular tissue. Furthermore, sEng can inhibit capillary formation in vitro and induce alterations in vascular permeability and hypertension in vivo. Acting in conjunction with sFlt1, sEng can cause severe PE and HELLP, as well as fetal growth restriction [70,71,72].

The imbalance between pro- and anti-angiogenic factors has been observed in mothers with PE, and its effects affect their offspring; however, its presence in offspring is not as clear. In the case of PGF, there is limited information about its fetal production. It has been documented that the expression of PGF/Pgf can occur in human and mouse oocytes, as well as in embryos from the stages of zygote to blastocyst [111,112]. This could be due to PGF being produced in other cell types, such as endothelial cells and hepatocytes, in addition to the placenta [113]. Furthermore, it has been observed that its production can be induced in adult neurons, Schwann cells, and astrocytes [114,115,116]. As PGF is a large glycoprotein, it is unlikely that the maternal plasma PGF crosses the placenta, although it can induce signaling through VEGFR1 or neuropilins expressed in the placenta [117]. A study reported that human amniotic fluid between weeks 7 and 9 contains approximately half the concentration of PGF compared to maternal serum [118]. Mid-pregnancy, the PGF and VEGF levels increase in amniotic fluid, with VEGF concentration six times higher than that of PGF [119,120]. In late pregnancy, PGF is generally undetectable in the amniotic fluid or umbilical cord blood of fetuses [96]. In 2016, Luna et al. sought to identify whether PGF is expressed in the developing mouse brain and contributes to its vascularization. They assessed the expression of Pgf/PGF, Vegfa/VEGF, vascular endothelial growth factor receptor (Vegfr)1, and Vegfr2 in the brains of normal C57BL/6 (B6) mice and Pgf^−/−^ mice on embryonic days (E) 12.5, 14.5, 16.5, and 18.5 to obtain the temporal–spatial expression of PGF during cerebrovascular development. Their findings indicated that Pgf/PGF and Vegfr1 are predominantly expressed in the prosencephalon at E12.5–14.5 compared to VEGF and Vegfr2. Vegfa/VEGF is more abundant in the rhombencephalon. PGF and VEGF expression was similar in the mesencephalon. Delayed vascularization of the rhombencephalon was observed at E10.5 and 11.5 in Pgf^−/−^ brains. At E14.5, the Willis circle of Pgf^−/−^ animals showed unilateral hypoplasia and fewer collateral vessels, defects that persisted after birth. Therefore, alterations in PGF expression in the mouse brain during gestation induce cerebral vascular defects that could lead to structural alterations in the CNS and its blood flow [103].

On the other hand, in mothers with PE, elevated concentrations of sFlt1 in serum have been identified [87,121], as well as lower concentrations of free VEGF and PGF [87]. After delivery, sFlt1 concentrations decrease rapidly, which could indicate a placental origin of sFlt1 during PE, but it is not entirely clear whether the fetus can contribute to the increase in sFlt1 [87,121]. Regarding this, Staff et al., in 2005, evaluated mothers with PE and healthy pregnancies (controls), taking samples of maternal serum, umbilical vein, and amniotic fluid to identify the concentrations of sFlt1, VEGF, and PGF. Their results show that the concentration of sFlt1 in PE conditions was higher than that in the control group, in the umbilical venous serum, maternal serum, and amniotic fluid. Furthermore, the concentration of PGF in the maternal serum was lower in the PE group compared to the control. The authors conclude that the concentration of sFlt1 is elevated in fetal circulation in preeclampsia but at a much lower level than in maternal circulation, which could suggest fetal contribution to the increase in sFlt1 concentration in preeclamptic pregnancy [96].

### 2.3. Inflammation

In PE, abnormal activation of the immune system has been observed, leading to an imbalance between the production of proinflammatory and anti-inflammatory cytokines, favoring a persistent inflammatory state [122,123,124]. The inflammatory state is associated with placental ischemia and systemic endothelial dysfunction, which characterizes the pathology and worsens as the pregnancy progresses [125,126].

This inflammation could induce neurodevelopmental alterations in PE-F1 [32,41,43,44,45,46,51]. In this regard, under conditions of placental ischemia, as in PE, an increased expression of interleukins such as IL-6, IL-17, IL-1β, IL-18, IL-4, IL-10, chemokines, and eotaxins (CCL11, LIX/CXCL5, and MIP-2/CXCL2) has been observed, leading to a neuroinflammatory environment [32,41,46]. About this, placental ischemia contributes to a dysfunction of the walls of cerebral vessels, facilitating the passage of interleukins through the placenta and into the fetal cerebral circulation [32,46]. Regarding this, in 2014, Martínez et al. indicated that placental ischemia could increase the incidence of brain microhemorrhages, and such microbleeds occur when there is sustained structural damage over time in the blood vessels [127]. One of the main causes of brain microhemorrhages is hypertensive vasculopathy because of prolonged exposure of the cerebral microvasculature to high blood pressure. Brain microhemorrhages can lead to long-term neurological damage, including cognitive and motor deficits, which could be another cause of the alterations observed in the offspring of preeclamptic mothers [127]. Giambrone et al., in 2019, induced placental ischemia in rats (via a surgical reduction in uteroplacental perfusion (RUPP)) and identified a higher number of cerebral microbleeds and proinflammatory cytokines in the E19-exposed fetuses, confirming the presence of microvascular dysfunction [46]. These vascular alterations due to microbleeds resulting from placental ischemia could be attributed to the hypoxic environment, confirmed by the authors based on the high hematocrit in the RUPP group [46]. On the other hand, we must not forget that the binding of sFlt-1 to free circulating VEGF and sEng to TGF-β1, along with a decrease in PGF production by trophoblasts, are to some extent responsible for the systemic endothelial dysfunction and placental inflammation related to PE [128].

Furthermore, interleukins such as TNF-α and IL-6 lead to an increase in vascular permeability, and TNF-α can activate endothelial cells, decrease the amount of mRNA in nitric oxide synthase, and increase the production of endothelin-1, which is a potent vasoconstrictor [129,130,131].

Considering that interleukins enhance processes such as inflammation, angiogenesis, proliferation, survival, differentiation, and neuronal function, they could be responsible for altering synaptic gene expression and enhancing nuclear factor-κB (NF-KB) and extracellular-signal-regulated kinase (ERK) signaling cascades, potentially implicated in the development of ASD through inhibiting gamma-aminobutyric acid (GABA) receptors [32].

IL-6 is secreted by tissues such as T cells, macrophages, osteoblasts, the smooth muscle cells of blood vessels, and the placenta, and its levels significantly increase in PE [109,132,133,134]. Elevated levels of IL-6 have been linked to an increase in microhemorrhages in the brains of PE-F1, which is associated with vascular damage in fetal brains, leading to long-term neurological damage and associated cognitive and motor deficits [46]. IL-6 and TNF-α increase in circulation and in trophoblast cells of the placenta [124], contributing to endothelial dysfunction through increasing adhesion molecules and the permeability of endothelial cells [135,136]. Furthermore, their elevated levels have been identified in studies related to autism in both animal and human models [137,138]. In animal models, it has been observed that IL-17a is activated in the fetal brain in response to maternal immune activation during PE. This is associated with behavioral alterations and an abnormal cortical phenotype in the offspring of mothers with PE [139]. It should be noted that the inflammatory, angiogenic, and apoptotic response and its cerebromorphofunctional correlate differ depending on the sex of the progeny [103,140,141,142]. Regarding this, Gumusoglu et al. (2021) identified that at E18 and P7, male offspring exposed to AVP had a smaller dorsal precerebrum, mainly in the intermediate, subventricular, and ventricular zones in males, along with specific sex anomalies in cortical and subcortical growth, synaptic protein distribution, and embryonic brain transcriptomics [5]. Regarding female offspring exposed to AVP, they showed decreased rotarod procedural learning compared to controls, which could be related to the evaluation of excitatory synapse density in memory-related brain areas: the hippocampus DG, CA1, and CA3 and the prefrontal cortex. This revealed a decrease in density in the DG only in female offspring exposed to AVP. Conversely, male offspring exposed to AVP exhibited increased anxiety-like behavior [32]. Furthermore, at P7, male offspring exposed to AVP had a decrease in caudate–putamen volume, while females had an enlargement of caudate–putamen volume relative to cortical volume. In adulthood, these deficits in males were resolved, but the same did not occur in females. This increase in caudate–putamen volume (neuronal substrate of procedural learning) would be related to procedural memory deficits in adult female offspring exposed to AVP, such as performance on the rotarod [32]. Carver et al., in 2014, also demonstrated that PE alters brain development in specific patterns according to the offspring’s sex and that prenatal pravastatin therapy prevents altered neuroanatomic programming in an animal model. Male offspring showed decreased volumes in the fimbria, periaqueductal gray, medial striata, and ventricles and increased volume in the lateral globus pallidus and neocortex. However, female offspring showed increased volume in the ventricles, medial striata, and retroflex fascicle and decreased volume in the inferior colliculus, thalamus, and lateral globus pallidus. Neuronal quantification via Nissl staining indicated a more pronounced decrease in cell count in the neocortex of male offspring, where prenatal pravastatin treatment ultimately prevented all these changes [140]. Rätsep et al. (2016) identified that mice genetically suppressing Pgf expression (as a PE genetic model) exhibited sexually dimorphic alterations in the adult brain anatomical structure, accompanied by impaired behavior and cognitive functions. These structural and functional brain alterations occurred in a sexually dimorphic pattern, possibly reinforcing the notion that male and female fetuses “allocate resources” differently in the uterus [49]. Similarly, Kay et al., in 2019, studied the behavior and neuroanatomy of adult Pgf^−/−^ mice and observed sexual dimorphism in offspring, specifically with depressive behavior [141]. These findings align with those expressed by Luna et al. (2016), who stated that PGF deficiency and PE in utero experience affect male and female brains differently [103]. Lastly, Valencia-Narbona et al. (2023) observed decreased expression levels of vascular endothelial growth factor in the cerebellar vermis of both sexes’ offspring. Additionally, they observed an increase in phosphorylated VEGF receptor 2 at Y951 in the male offspring and a decrease in locomotor learning in the female offspring of preeclamptic mothers [91].

### 2.4. Disruption in Neurons, Glia, and Neuronal Signaling in PE-F1

During PE, the development of the fetal brain’s nervous tissue is disrupted [48], mainly due to a decrease in the levels of nerve growth factor (NGF) in the maternal plasma, as well as a reduced expression of cAMP response element-binding (Creb), histone-acetyltransferase (Ep300), and Fibroblast Growth Factor-2 (FGF-2) in PE-F1 [39], all of which are essential for the growth of neuronal cells [50]. It has also been demonstrated that these factors play crucial roles in hippocampal neurogenesis, a structure that is fundamental for spatial learning and memory [39]. Additionally, there is evidence suggesting a disruption in neuronal circuits, which may also be related to memory deficits in PE-F1 [32].

The number and length of astrocytes are increased in the offspring of mothers with PE [39,42], which may occur in order to recover the weight and size of the brain [39]. Regarding this, Liu et al. (2016) observed an increase in gliogenesis to compensate for offspring brain weight at P56 in an L-NAME group of a PE model. Although brain weight and laminar structure were essentially normal at P56, spatial learning and memory were severely affected. Additionally, they detected a deficiency in neurogenesis contributing to smaller brains in the L-NAME group at P0, concluding that lower brain weights and smaller brain sizes may have resulted from a deficiency in neural progenitor cell proliferation in the early development stages without affecting the differentiation of neural progenitor cells or radial glial scaffold morphology. As glial cells constitute nearly 50% of mammalian brain cells and astrocytes, which proliferate after birth, are the largest glial cell population, researchers explored the neuron and astrocyte numbers in adult brains via immunostaining for the neuronal marker NeuN and the astrocytic marker GFAP. Interestingly, they found that the number of GFAP+ cells increased throughout the adult L-NAME group brain after the reduction in NeuN+ cell numbers. These data suggested to them that astrocyte proliferation may contribute to offspring brain weight recovery in mothers with PE and that the hippocampal neurogenesis impairment in adult offspring that they observed (along with the reduced expression of Creb, Ep300, and FGF-2) is related to the demonstrated cognitive deficiency. However, they did not rule out that dysregulated astrocyte generation in the hippocampal dentate gyrus region may also modulate spatial learning alterations and defective memory detected in the water maze test. This is because astrocytes are described as crucial for regulating connectivity and synaptic function [39].

A decrease in dendrite length and in the length of tyrosine hydroxylase-positive (TH+) processes, an increase in GABA receptors, and a reduction in glutamate receptors (GLUN1) have also been identified [32,42]. Regarding this matter, Scott et al. (2018) suggested that the placenta plays a key role in the dysfunction of the neural circuitry characterizing offspring neurodevelopmental disorders, including a potential imbalance of excitatory and inhibitory factors. To identify this imbalance, the authors analyzed (in vitro) whether placental tissue released from mothers with PE secreted molecules that could affect cultured cortical cells, discovering that applying a PE placental culture medium to mixed cortical cultures caused changes in neurons and astrocytes, which is related to key changes observed in the brains of patients with schizophrenia and autism. They observed effects on dendrite lengths and the number of astrocytes, as well as on glutamate levels and γ-aminobutyric acid receptors. Additionally, they identified a bidirectional communication requirement between neurons and astrocytes, potentially through glutamate, to produce the effects of preeclamptic placenta on cortical cells, causing anomalies in cortical neurons and astrocytes as well as the process lengths of tyrosine hydroxylase-positive neurons (TH+), suggesting that glutamate could also play a role in mediating the harmful effects of the PE medium on neurons. Overall, the changes in the astrocytic process lengths and glutamate receptor subunit levels observed in mixed cortical cultures after PE medium exposure were not replicated in neuron-only cultures, suggesting that the presence of non-neuronal cells was truly necessary for the preeclamptic medium to affect the neuronal culture. Altogether, the results suggest that the presence of neurons may be necessary for the PE medium to exert its effect on astrocyte process length but not on the number of astrocytes [42].

Therefore, bidirectional communication between astrocytes and neurons is vital during neurological development, participating in neuron migration, survival, and synapse maturation and thereby promoting the proper functioning of neuronal circuits. Astrocyte–glutamate communication, in turn, plays a fundamental role in synaptic function, as astrocytes can modulate synapses through releasing glutamate. On the other hand, neurons influence the typical morphology of astrocytes, along with their gene expression. In this context, astrocytes play a significant role in neuronal damage in PE models, as the presence of astrocytes mediates a reduction in GLUN1 levels. This suggests that astrocyte–glutamate signaling is involved in the changes observed in neurons [42].

In the study of Gumusoglu et al. (2021), through a model generating a preeclamptic phenotype via prenatal vasopressin (AVP) exposure in pregnant mothers, the authors demonstrated that both female and male offspring at postnatal day 7 (P7) had smaller neocortices. Conversely, adult males exposed to AVP exhibited reduced total neocortical volumes [32]. In contrast with the findings of Scott et al. (2018) [42] and Liu et al. (2016) [39], this study showed no changes in adult cortical volume, total cell density, neuronal density, or macroglial densities (astrocytes and oligodendrocytes) in either male or female offspring exposed to AVP. Similarly, the volumes of the prefrontal cortex, corpus callosum, and hippocampus (DG and CA) remained unchanged. However, procedural learning was disrupted in the female offspring. Prior studies on the effect of gestational vasopressin administration in rat offspring behavior had also revealed memory-related process alterations, including reduced memory retrieval in male offspring. Gumusoglu et al. (2021) evidenced cognitive impairment in the offspring of mothers with a preeclamptic phenotype (due to prenatal AVP exposure). A mechanistic evaluation suggested that these anomalies may not be mediated by astrocyte alterations. It is noteworthy that the methods of inducing preeclampsia in these three studies differed. Liu et al. (2016) induced PE in rodents via prenatal L-NAME administration to pregnant mothers. Scott et al. (2018) examined whether placental factors damaged neuronal and astrocytic cultures, while Gumusoglu et al. (2021) induced the preeclamptic phenotype through AVP administration, showing discrepancies in their results compared to previous studies, with differences in the AVP administration methods. These collective evaluations could lead to critical mechanistic insights into specific treatments and/or preventions of PE neurodevelopmental outcomes in clinical populations. However, further studies investigating the underlying neuronal and glial molecular mechanisms of cognitive impairment observed in the offspring of mothers with PE are needed.

Consistent with the above, evidence indicates that neurogenesis is disrupted in PE-F1 [32,39,45,46,49], and it could be one of the triggers for a disruption in the development of neuronal circuits observed in the offspring of mothers, with PE affecting the proper functioning of the CNS in PE-F1 [32,50].

In this context, an increase in the expression of GABA receptors has been associated with the onset of schizophrenia [59], epilepsy [42], and ASD [32,42]. Similarly, a reduction in GLUN1 receptors has been observed in association with ASD in PE-F1 [42]. However, the established associations in the evidence between PE and neurodevelopmental disorders such as ADHD, CP, intellectual disability [40,47], and reduced cognitive ability [50] do not allow for the establishment of direct causal relationships between these pathophysiological mechanisms and any of the neurodevelopmental alterations.

## 3. PE and Neurological Alterations in the Offspring

**Figure 2:**
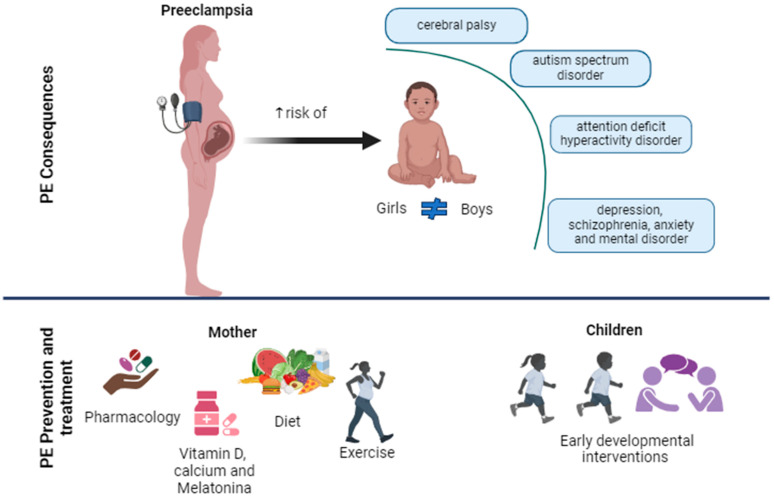
Neurological alterations in the offspring of mothers with PE and potential tools for prevention and treatment for pregnant mothers and their children. The figure shows potential neurological alterations in children associated with PE in mothers, such as cerebral palsy, autism spectrum disorder, attention deficit hyperactivity disorder, anxiety and mood disorders, schizophrenia, and emotional dysregulation. Additionally, it is noted that these alterations may vary depending on the gender of the child. Possible preventive interventions for PE in mothers include diet, exercise, pharmacological treatment, and vitamin use. For children, an effective treatment tool could be stimulation in the first years of life. Abbreviations: PE, preeclampsia; ↑, increase.

There is evidence that could link the mother’s PE to a higher risk of neurodevelopmental alterations in the offspring, such as CP [32,34,143], ASD [32,144,145], ADHD [32,145,146], anxiety and mood disorders, schizophrenia, emotional dysregulation, and eating disorders [32]. Next, we review this evidence for more complex neurological alterations such as CP, ASD, and ADHD (Figure 2).

### 3.1. PE and CP

Various authors have indicated a correlation between PE and CP, noting that children exposed to PE had a 2.5 times higher risk of developing CP compared to non-exposed children [34,147,148]. In early-onset PE, the risk of developing CP is even higher compared to mothers with late-onset PE [148]. Children exposed to PE with a gestational age ≥ 37 weeks and appropriate size were not associated with a higher risk of CP. On the contrary, children born with small gestational age (SGA) have a significantly higher risk of developing CP [34]. Similarly, newborns (NBs) of preeclamptic mothers may experience birth asphyxia and have a four times greater likelihood of being born SGA, having lower birth weight, and having Apgar scores less than 5 at 1 and 5 min compared to those born to normotensive pregnancies [148]. On the other hand, PE appears to predispose to the development of spastic CP with unilateral distribution in full-term infants, possibly due to a perinatal ischemic stroke influenced by the characteristic vascular endothelial disorder of PE [34]. Likewise, the systemic inflammation characteristic of PE, similar to sepsis, could also contribute to the onset of CP [34,149].

Therefore, PE exposes the offspring to a higher probability of developing CP in full-term gestational-age deliveries [148]. However, this association becomes even more significant as the neonate’s gestational age decreases [150]. Furthermore, the combination of prematurity and intrauterine growth restriction (IUGR) predisposes the baby to a higher risk of CP, especially in the case of complications such as infection and perinatal asphyxia [34,148].

### 3.2. PE and ADHD

Various authors have correlated PE with an increased risk of ADHD in offspring, independent of gestational age at birth [139,151,152,153,154]. However, other authors do not support this correlation [144,155]. In this regard, Mann et al. proposed that the combination of PE and IUGR increases the probability of ADHD by 43%, similar to PE alone [153]. Likewise, it has been described that children of Caucasian descent have a higher risk of ADHD while being female has been described as a protective factor, likely due to their higher levels of oxytocin, among other factors [153,154]. In 2014, Silva et al. suggested that administering oxytocin during childbirth had a neuroprotective effect on newborn girls, although they acknowledged the possibility of this finding being coincidental [154]. In a 2012 case study by Kosaka et al., a 16-year-old girl with ADHD showed significant improvements in social interactions and communication and a reduction in irritability and aggressive behavior after receiving an oxytocin nasal spray for two months [156]. Conversely, research on boys revealed a negative correlation between serum oxytocin levels and ADHD scores, alongside a positive correlation with empathy [157,158]. Demirci’s study highlighted that, among ADHD subtypes, the inattentive subtype exhibited notably higher serum oxytocin levels compared to the hyperactive/impulsive subtype, which is more prevalent and associated with higher aggression risk and lower empathy in boys compared to girls [158]. However, further research is warranted to explore the sex-specific effects of oxytocin on ADHD and its underlying mechanisms related to social memory, attachment, and trust [159].

Similarly, infants exposed to early severe PE have an even greater risk of ADHD compared to infants exposed to mild PE [144]. A cohort study by Maher et al. in 2020 showed that PE was associated with a 15% increase in the probability of developing ADHD. In the case of an IUGR newborn exposed to PE, the probability of having ADHD increases to 43% and reaches 55% in the case of twins [139]. Additionally, the children of mothers with PE and genitourinary infection showed a 53% probability of having ADHD compared to children not exposed to any alterations during pregnancy [139].

Fetal exposure to conditions of hypoxia and ischemia could promote the development of ADHD due to the occurrence of structural and functional brain injuries that occur during a critical period of organ development [160]. A study by Zhu et al. in 2016 using mice revealed that the density of dendritic spines of pyramidal neurons in the CA1 region of the hippocampus was significantly lower in the group with brain injury from hypoxia–ischemia triggered by PE compared to control cases of offspring from mothers without PE [160]. Along with this, a poor microstructural integrity of the CA3 region of the hippocampus was observed, where mice exposed to these conditions also showed cell death through the astrogliosis of oligodendrocyte progenitor cells, excessive apoptotic cell death of subplate neurons, and activation of microglia in the frontal cortex [160]. Furthermore, the animals also exhibited cognitive deficiencies in task performance, deficits in sustained attention, and increases in impulsivity and compulsivity in response to manipulation tasks, which correlated with a loss of brain volume throughout the hemisphere, cerebral cortex, white matter, hippocampus, and striatum [160]. Previous evidence from imaging studies in babies with hypoxia–ischemia demonstrated a marked reduction in absolute gray matter volume, intraventricular volume, and the appearance of periventricular leukomalacia (PVL) [161,162], which could predispose to the development of ADHD.

### 3.3. PE and ASD

Prenatal exposure to PE confers a significant risk factor for the onset of ASD in offspring, regardless of the trimester in which the diagnosis was established, with a percentage ranging from 25% to 32% higher risk of developing ASD compared to offspring not exposed to PE [139,144,163,164,165,166,167,168]. A stratified analysis of the duration of PE exposure demonstrated that children exposed to PE for 2 or more days had a significantly higher risk of ASD compared to non-exposed children. On the contrary, exposure for less than 24 h showed no association with the risk of ASD [164,166,168,169].

Assessing the functional abilities of children in three areas—activities of daily living, mobility, and social/cognitive—using the Pediatric Evaluation of Disability Inventory-Computer Adaptive Test (PEDI-CAT), it was found that children born from preeclamptic pregnancies scored lower in the social and cognitive domain than in other areas of the test [170].

Pathologies or placental abnormalities that manifest during the third trimester or near the time of delivery can have direct effects on the rapidly developing fetal brain. This is mainly due to the disruption of blood flow to the fetus as well as the generation of OS, leading to damage and dysfunction of developing neurons [163,166,169,171]. In this regard, the brain is highly vulnerable to OS due to its limited antioxidant capacity, micronutrient deficiency, and metabolic dysfunction, which are potential biological mechanisms explaining the link between PE and the increased risk of ASD in offspring [163].

Maternal inflammation may represent an underlying mechanism in the programming of offspring’s neurodevelopment, affecting connectivity and functional and cerebral development, impacting cognitive levels, and contributing to the onset of ASD [164,172,173]. Similarly, inflammation within the blood vessels of the chorionic plate and poor maternal vascular perfusion are strongly associated with a higher risk of ASD in male offspring but not in females [174]. Regarding these sex differences, Xie et al. (2020) define ASD in a sexually dimorphic manner when the triggering factor is PE. One of the potential molecular triggers for these sex differences is the RPS4Y1 gene encoded by the Y chromosome, an inhibitor of STAT3 signaling, which qualifies as a possible contributor to the male predominance of ASD [175]. A study by Straughen et al. in 2017 determined that inflammation within the vessels of the chorionic plate and poor maternal vascular perfusion affecting placental shape are strongly associated with an increased risk of ASD in male offspring but not in females [171]. Furthermore, the authors described that the presence of edema in placental villi is related to a lower risk of ASD in males [171]. Another possible explanation for the sex differences in neurological developmental disorders in offspring of preeclamptic mothers could be alterations in placental insulin receptor (InsR) signaling [176,177]. In this regard, male transgenic mice with conditional InsR deletion in fetal trophoblasts exhibit increased HPA stress responses, as well as deficits in sensorimotor activation, unlike females. Probably, in males, changes in gene expression in the placenta are related to vascular function, amino acid transport, 5-HT homeostasis, and mitochondrial function, presenting transcriptomic differences that suggest impaired cortical development. Another possible mechanism that could determine sex differences in the placental response may be differences in DNA methylation or other epigenetic changes [178,179]. In this regard, placental miR-34a, miR-146a, and miR-222 are positively associated with telomere length in female placentas but not in males, which protects chromosome ends from damage [180]. Males gestated in a stressful maternal environment, such as in PE, develop maladaptive stress responses and deficits in the hypothalamic–pituitary axis (HPA). Additionally, in male mice subjected to prenatal stress, increased proinflammatory cytokines (Il6 and Il1b) in the placenta, stress-induced locomotor hyperactivity, and alterations in the neuronal expression of DA, D1, and D2 receptors have also been observed [181]. Therefore, maternal stress due, for example, to PE, induces sex-dependent differences in placental responses. Male mice show greater vulnerability to maternal stressors, increasing the risk of neurobehavioral deficits such as ADHD in the future. However, further studies are needed to identify the various causes that increase the incidence of ADHD in male offspring when it occurs as a result of PE.

Clinical and preclinical studies have shown that maternal inflammation and hypoxia produce marked structural changes in the brain that could be consistent with some neuroanatomical characteristics of ASD [144,164,165,166,168,169,172]. This can result in over- or under-growth of brain volume, increased microglial density, reduced arborization of the cerebellum and cortex, and a reduced number of Purkinje cells and pyramidal layer in the dorsal regions of the hippocampus [144,164,166,168,169,172]. For example, through magnetic resonance imaging (MRI), it was observed that the brains of children with ASD exposed to PE showed higher regional volumes in the cerebellum, brainstem, temporal lobe, and the right and left amygdalae [49,167]. These children also exhibited a trend towards a lower ratio of cerebral blood vessels, mainly in the occipital and parietal lobes [49,167].

Furthermore, PE commonly causes a decrease in maternal immunoglobulin G (IgG), which is intimately related to the onset of ASD, and the appearance of behavioral symptoms is associated with the presence of maternal anti-fetal brain antibodies [170]. It has also been observed that certain maternal cytokines, such as IL-6, can traverse the placenta and enter fetal circulation, potentially modulating neuronal proliferation, survival, differentiation, and function [41]. In addition, Kim et al. (2019) describe that the inflammation and insulin resistance occurring in maternal metabolic syndrome (chronic hypertension, gestational hypertension, preeclampsia, and overweight) are associated with an increased risk of autism spectrum disorder in the offspring. This syndrome alters the immune and metabolic systems, generating a stressful gestational environment that determines long-term fetal outcomes [164].

The placenta produces neurotransmitters such as serotonin and catecholamines that are intimately related to intrauterine circulation and brain function [174]. Specifically, serotonin (5-HT, 5-hydroxytryptamine) stimulates cell division, neuronal migration, cell differentiation, and synaptogenesis [76]. Thus, alterations in the placental production of 5-HT increase the risk of ASD because an elevation of this neurotransmitter will inhibit the production of oxytocin by the paraventricular nucleus (PVN) of the hypothalamus. This, in turn, enhances calcitonin gene-related peptide (CGRP) in the central nucleus of the amygdala. Both promote social behaviors, and disruption of these processes can lead to neurobehavioral disorders affecting sensory, motor, and cognitive abilities [174]. Regarding placental morphology, it has been observed that PE, due to decreased uteroplacental perfusion, contributes to the appearance of bi-layered folds in abnormal trophoblasts, which has been associated with the development of ASD [182].

### 3.4. PE and Other Neurological Alterations

As mentioned earlier, the chronic placental insufficiency present in PE has the potential to influence fetal cerebral perfusion and to have long-term effects on brain development [183,184]. Episodes of placental hypoxia or reperfusion induce OS and the excessive systemic inflammatory response of PE, directly impacting fetal brain growth [183,184]. In this context, increased OS in the mother may have more significant consequences for the growth and development of male infants, not only during pregnancy but also during the early stages of postnatal life [185]. An observational study by Girchenko et al. in 2020 mentioned that the offspring of mothers exposed to high levels of inflammatory biomarkers (hsCRP and acetyl-glycoprotein) were associated with larger areas of delayed infant neurological development in a 10.8-year follow-up [172]. Additionally, through favoring cognitive impairment in offspring, PE contributes to a lower intelligence quotient (IQ) in these offspring [49].

Similarly, an observational study analyzed the effect of PE on neurobehavioral outcomes in late preterm infants (greater than 32 weeks) using the Neurobehavioral Assessment of the Preterm Infant (NAPI) score, which measures the progression of neurobehavioral performance [183]. The average NAPI score for motor–vigor development (MDV) and alertness–orientation (AO) was lower in the group of children born to mothers with PE compared to controls, indicating that newborns of mothers with PE exhibit immature neurological behavior [183].

However, mild PE is not associated with such adverse outcomes in the neurodevelopment of offspring. In contrast, infants born to mothers with severe PE were more likely to experience failure in at least one category of the Ages and Stages Questionnaire (ASQ), indicating psychomotor development delay up to 3 years inclusive [168]. The ASQ evaluates aspects such as gross motor skills, fine motor skills, communication, personal social skills, and problem-solving [168].

### 3.5. Sexual Dimorphism Described in the Alterations Caused by PE in the Offspring

It has been reported that, during preeclamptic pregnancies, notable differences in angiogenic, vascular, neuronal, and functional brain responses exist, contingent on the fetal sex [91,186,187,188]. In this regard, there is a discernible variation in the impact of exposure to PE based on the sex of the offspring.

Several authors have asserted that, in preeclamptic pregnancies, the inflammatory, angiogenic, and apoptotic responses, along with their cerebromorphofunctional correlates, exhibit disparities contingent upon the sex of the progeny [24,103,140,141]. Valencia-Narbona et al. (2023) have established that the placenta exhibits significant sexual dimorphism, potentially contributing to the origin of the observed alterations. Moreover, their findings identified the brain as being structurally sexually dimorphic and vascularly dimorphic, as other authors have described [91,189,190]. For example, Gumusoglu et al. (2021) have shown these diverse gene expression patterns. Their results showed differential expression in various genes, encompassing embryonic gene expression in the offspring, placental gene expression, and the intricate dynamics of cortical and subcortical growth. Moreover, their work extends to the distribution of synaptic proteins, providing a comprehensive understanding of the intricate molecular processes shaping neurodevelopment [32]. Similarly, cognitive function is more disrupted in the male offspring of preeclamptic rats compared to the female offspring of rats with PE [187,188].

In human studies, the sex-specific effects of PE exposure on neuropsychiatric outcomes in children remain uncertain. For example, some studies have suggested that male offspring may exhibit increased resilience to psychiatric illnesses or mood disorders following exposure to PE [35]. Nevertheless, other research indicated more pronounced cognitive impacts on males decades after exposure to PE [191].

Therefore, the sex of the progeny could be a confounding factor in the interpretations of offspring alterations due to PE, if not studied differentially. Additionally, the exact role of sex as a biological variable in the programming of neurodevelopmental exposure to PE will require more in-depth clinical and preclinical studies [192].

## 4. Prevention and Treatment of PE in Pregnant Mothers and Their Offspring

At present, the treatment of choice for PE primarily focuses on relieving symptoms and complications for the mother and/or fetus [193] (Figure 2). Essentially, it involves monitoring, periodic blood pressure checks, preconception counseling, and the use of medications that can reduce blood pressure [194]. However, the American College of Obstetricians and Gynecologists does not recommend treatment with such antihypertensive drugs for patients with mild to moderate hypertension (systolic < 160 mmHg or diastolic < 110 mmHg) during pregnancy, as this specific treatment may not decrease the risk of disease progression, and there is evidence suggesting a potential association with the occurrence of small-for-gestational-age newborns in preeclamptic pregnancies [195].

Another treatment option for PE is melatonin (N-acetyl-5-methoxytryptamine), an endogenous antioxidant secreted by the pineal gland and the placenta during normal pregnancy [196]. It is known that melatonin levels are decreased in preeclamptic pregnancies [197,198], probably due to the reduced expression of the enzymes synthesizing melatonin (arylalkylamine N-acetyltransferase and hydroxyindole-O-methyltransferase) in placental tissue as well as the decreased expression of melatonin receptors (M1 and M2) [199]. Through supplementation, melatonin has been observed to have antioxidant and antihypertensive effects and is an important alternative in the treatment of PE [200]. Its vasoactive effects have been evidenced in animal models [201,202] and in humans, with an observed hypotensive effect that would favor a lower maternal blood pressure, delay labor, and avoid premature birth [203]. Regarding this, melatonin could have benefits not only in the pregnant mother but also in the fetus as a protector of the fetal brain in a hostile intrauterine environment, such as in PE [200,204].

Non-pharmacological management approaches used in preeclamptic mothers include diet and exercise before and during pregnancy. Regarding diet, the presence of fruits and vegetables, as well as legumes, dairy products, nuts, and tubers, among others, has shown favorable results in preventing PE [205,206,207,208]. Another preventive element is supplementation with vitamin D [209] and calcium [210].

Regarding exercise and its effect on mothers with PE, evidence suggests that exercise reduces the risk of PE [207,211,212,213,214,215,216,217] and has been shown to reduce the risk of PE by up to 40% [212,213], preventing and/or mitigating vascular consequences in hypertensive disorders (including PE), whether performed during pregnancy or before. However, the reduction in the risk of PE incidence is greater if exercise is performed before 24 weeks of gestation [217]. Nevertheless, it is challenging to identify the type of exercise and its most suitable characteristics for mothers with PE due to the various intensities, dosages, and types of exercise applied in research, which currently hinders a consensus on the best option and its outcomes.

Regarding the children of mothers with PE, it is known that early childhood—especially the first three years of life—is a critical stage of brain development, impacting both cognitive and social aspects of an individual [218,219]. Both adverse and favorable events experienced during this period can influence a child’s neurodevelopment [220,221]. In this regard, we have extensively described the mechanisms and consequences in the child of a preeclamptic environment during gestation. To address this, some measures can be applied to mitigate the consequences of PE on neurodevelopment. Early developmental interventions or early stimulation can be crucial tools in this regard; when performed appropriately, based on the child’s age and receptive attention, early developmental interventions and early stimulation have been observed to provide benefits to the child’s brain architecture, strengthening and pruning synaptic structures and connections [219,222,223] (Figure 2). Early developmental interventions or early stimulation programs promote the development of premature infants in terms of physical, mental, and social aspects [224]. These interventions are based on the application of physical, psychological, and baby-centered education therapy, focusing on the parent, the child, or the parent–child relationship, as well as on the social interaction between infants [224]. Their goal is to reduce physical and mental development issues in premature children and infants through activities that support and enhance their development [224]. This form of stimulation is part of a family-centered approach, encouraging both child and parent or caregiver participation, thus fostering the child’s confidence during the intervention [224,225], and corresponds to an intervention that can prevent future developmental delays in premature infants [226].

In this context, early developmental interventions have been applied to animal models, referred to as an enriched environment (EE). This corresponds to an intervention involving the stimulation of the offspring through physical exercise, sensory information, and social stimulation. It has been observed that enriched environments are capable of inducing neuroplasticity and recovering behavioral alterations [227,228,229,230,231]. Exposure to an EE could enhance neurogenesis and cell survival; increase the branching, length, and number of dendritic spines; and regulate neuronal growth factors [232,233,234,235]. This beneficial effect may stem from changes in transcription and gene expression, protein interactions, and/or the levels of proteins involved in the neuronal structure and the formation of additional synapses [229,236,237,238,239,240,241], which can induce synaptic plasticity at the sensory, motor, and associative levels [229]. Strengthening neuronal connectivity through an EE may promote the efficient use of existing neural networks and the recruitment of alternative networks when needed, potentially leading to improved brain function and behavior [239]. Therefore, an EE can serve as a powerful intervention for adverse brain programming, being a fundamental strategy for managing neurodevelopmental alterations during high-risk pregnancies such as PE. It is expected that an EE has similar effects at the brain level as early developmental interventions in children.

Continued research on prevention tools and treatments for PE is necessary in order to avoid negative effects in both the mother and the child.

## 5. Conclusions

PE is a serious condition that has negative effects in both the mother and the fetus. These effects primarily stem from a disturbance in the placental vascular endothelium, directly affecting normal uteroplacental blood flow. This, coupled with the proinflammatory environment at the maternal/fetal level, the imbalance between angiogenic and antiangiogenic factors, and the hypoxic–ischemic environment, collectively creates an adverse environment for the fetus. This adversely impacts the normal development of the CNS, predisposing the offspring to neurodevelopmental disorders such as ADHD, ASD, and CP, among others.

Importantly, the pathophysiological mechanisms associated with PE that can impact neurodevelopment in the offspring of preeclamptic mothers include hypoxia; the dysregulation of vascular and angiogenic processes; inflammation; the disruption of neurons, glia, and neuronal signaling; and neurological alterations. These mechanisms are driven by the described alterations in the regulation of placental pro- and anti-angiogenic factors, such as vascular endothelial growth factor, placental growth factor, soluble fms-like tyrosine kinase 1 receptor, and soluble endoglin, among others. Notably, the alterations observed in the offspring of preeclamptic mothers highlight the relevant sexual dimorphism described at various levels.

It is essential to continue researching the pathophysiological mechanisms of PE and its connection to neurodevelopmental consequences in exposed children.

Additionally, exploring potential treatments and preventive interventions is crucial to mitigate the effects of PE in both mothers and children.
